# Venom Use in Eulipotyphlans: An Evolutionary and Ecological Approach

**DOI:** 10.3390/toxins13030231

**Published:** 2021-03-22

**Authors:** Krzysztof Kowalski, Leszek Rychlik

**Affiliations:** 1 Department of Vertebrate Zoology and Ecology, Institute of Biology, Faculty of Biological and Veterinary Sciences, Nicolaus Copernicus University in Toruń, 87-100 Toruń, Poland; k.kowalski@umk.pl; 2 Department of Systematic Zoology, Institute of Environmental Biology, Faculty of Biology, Adam Mickiewicz University in Poznań, 61-614 Poznań, Poland

**Keywords:** *Blarina*, food hoarding, intraspecific competition, *Neomys*, mammalian venom, prey hunting, shrews, *Solenodon*, toxicity, venom functions

## Abstract

Venomousness is a complex functional trait that has evolved independently many times in the animal kingdom, although it is rare among mammals. Intriguingly, most venomous mammal species belong to Eulipotyphla (solenodons, shrews). This fact may be linked to their high metabolic rate and a nearly continuous demand of nutritious food, and thus it relates the venom functions to facilitation of their efficient foraging. While mammalian venoms have been investigated using biochemical and molecular assays, studies of their ecological functions have been neglected for a long time. Therefore, we provide here an overview of what is currently known about eulipotyphlan venoms, followed by a discussion of how these venoms might have evolved under ecological pressures related to food acquisition, ecological interactions, and defense and protection. We delineate six mutually nonexclusive functions of venom (prey hunting, food hoarding, food digestion, reducing intra- and interspecific conflicts, avoidance of predation risk, weapons in intraspecific competition) and a number of different subfunctions for eulipotyphlans, among which some are so far only hypothetical while others have some empirical confirmation. The functions resulting from the need for food acquisition seem to be the most important for solenodons and especially for shrews. We also present several hypotheses explaining why, despite so many potentially beneficial functions, venomousness is rare even among eulipotyphlans. The tentativeness of many of the arguments presented in this review highlights our main conclusion, i.e., insights regarding the functions of eulipotyphlan venoms merit additional study.

## 1. Introduction

### 1.1. Natural Toxins

The dynamic development and application of molecular techniques to the study of venom (referred to as venomics; [1,2,3,4,5]) during recent decades have identified and characterized many natural toxins [5,6,7] and have offered a great opportunity for their use as pharmacological tools. In fact, many bioactive molecules have found applications in medicine and development of new drugs [8,9,10]. However, despite comprehensive studies on toxic molecules, including their purification and descriptions of their physiological and pharmacological mechanisms, our knowledge of the ecology and evolution of natural toxins is scarce. It is estimated that there are twenty million natural toxins in the world; however, only ten thousand have been identified, with ca. one thousand having been examined thus far [11]. It is not surprising that most studies have focused on biochemical analyses of animal venoms, due to their potential medical applications and severe human morbidity and mortality caused by snake bites or scorpion stings [12,13,14]. However, to fully understand the evolution of animal venoms, it is necessary to examine their biological functions and toxic effects on their natural targets (wild prey and/or enemies) instead of commonly used models such as laboratory mice, rats, rabbits or humans. Ecological studies focusing on natural, predator–prey interactions and using venom in prey hunting, competition, and avoiding predation, parasites and pathogens can also shed new light on the evolution of animal venom systems [15,16,17].

### 1.2. Venom Definition

As there is no single definition of venom nor venomous animal, major difficulties in classifying animals as venomous or nonvenomous may arise [18]. Thus, the number of recognized venomous animals may differ depending on the accepted definition. Bücherl [19], for instance, proposed three criteria that must be met to classify an animal as venomous: such animals must possess (i) at least one venom gland in which toxins are produced, (ii) a mechanism for venom excretion or extrusion, and (iii) a venom apparatus to inflict wounds and administer the venom into a target animal. Mebs [20] also states that venomous animals produce toxins in a group of cells or specialized venom glands, and possess the venom apparatus (e.g., fangs, stings, modified teeth, spikes, spurs, pincers and others), connected to the venom glands, to deliver the venom into target prey and/or a predator during a bite or sting. Additionally, toxic substances injected into the body of a target animal must disrupt its normal physiological processes and/or cause its death. Fry et al. [21] proposed a broader definition of venom to include animal taxa that have not previously been regarded as venomous by traditional definitions. Amongst these new venomous taxa are vampire bats, fleas and ticks [22], whose venom does not kill the prey but only enables feeding. Thus, Fry et al. [21] define venom as “a secretion, produced in a specialized tissue (generally encapsulated in a gland) in one animal and delivered into a target animal through the infliction of a wound. Venom must further contain molecules that disrupt normal physiological or biochemical processes so as to facilitate feeding or defense by/of the producing animal”. According to this definition, venomous animals do not have to possess a specialized venom apparatus to deliver toxins to the target [21]. Moreover, the same authors criticize the anthropocentric point of view of assessing venom toxicity solely based on toxic effects on humans or laboratory animals. As animal venoms did not evolve to kill humans nor laboratory animals the observed effects may not reflect the true toxicity of venom. Analysis of venom toxicity on wild taxa (natural target animals) and studies on ecological functions of venom and predator–prey interactions will thus likely be of fundamental importance to expand our understanding of the evolution of animal venoms.

### 1.3. Venomous Mammals

For centuries, the proposition that some mammals were venomous (similarly to snakes and spiders) was neglected by the scientific community and treated as folklore [23]. Recently, however, after discovering several extinct and arguably venomous taxa of the order Eulipotyphla [24,25,26,27,28,29], and the development of venomics [30,31,32,33], discussion about venomous mammals reopened. Nonetheless, in comparison to venomous insects, arachnids and reptiles, venom production in mammals is rare [34,35,36]. According to the traditional definition of venom, venomous mammals belong to only two orders: Monotremata with the platypus, *Ornithorhynchus anatinus* [37,38,39,40,41], and Eulipotyphla (formerly Soricomorpha) with two venomous *Solenodon* species and a few shrew species [34,35,36,42,43,44,45]. However, following the venom definition proposed by Fry et al. [21], three species of vampire bats (Chirpotera), i.e., *Desmodus rotundus*, *Diaemus youngi* and *Diphylla ecuadta* [22,35,46,47,48,49], and as many as eight species of slow lorises (*Nycticebus* spp.) (Primates) can be classified as venomous, although among lorises only four species (*N. bengalensis*, *N. coucang*, *N. pygmaeus* and *N. javanicus*) have been confirmed as venomous so far [16,35,50,51,52,53]. Additionally, hedgehogs, closely related to shrews [54], have been suspected of being venomous, but studies by Mebs [55] on the biological and enzymatic activities of saliva of the European hedgehog *Erinaceus europaeus* have shown that hedgehogs do not produce venom in their salivary glands. There is no doubt, however, that further paleontological, biochemical and ecological studies will extend the list of venomous mammals.

### 1.4. Purpose of This Review

Although advanced proteomics and genomics techniques are easily available, biochemical studies on mammalian venoms are restricted due to low quantities of secretions produced in venom glands, difficulties in maintaining some mammals, particularly shrews, in captivity, and the threatened status of numerous venomous mammals (shrews, solenodons) [16]. Thus, our knowledge on composition and toxicity of eulipotyphlan venoms is still scarce [34,35,36]. Among five recognized and 18 arguably venomous species, only venom of three of them have been characterized so far. In 2004, Kita et al. [30] identified and characterized blarina toxin from saliva of the short-tailed shrew, *Blarina brevicauda*. Recently, Kowalski et al. [43] described toxic proteins and physiological activity of venom of the Eurasian water shrew, *Neomys fodiens*, whereas Casewell et al. [44] characterized the profile and toxicity of venom from the Hispaniolan solenodon, *Solenodon paradoxus*. Thus far, most studies have focused on the biochemical characterization of venoms and their possible pharmacological applications [56,57]. However, to understand how eulipotyphlan venoms evolved, it is important to study their ecological functions and effects on their natural targets. In this review, therefore, we summarize venomous eulipotyphlans (and other putatively venomous species), describe biochemical profiles and toxic activities of their venoms, and discuss their biological adaptations in an evolutionary and ecological context. 

## 2. Extant Venomous Eulipotyphlans

**Figure 1:**
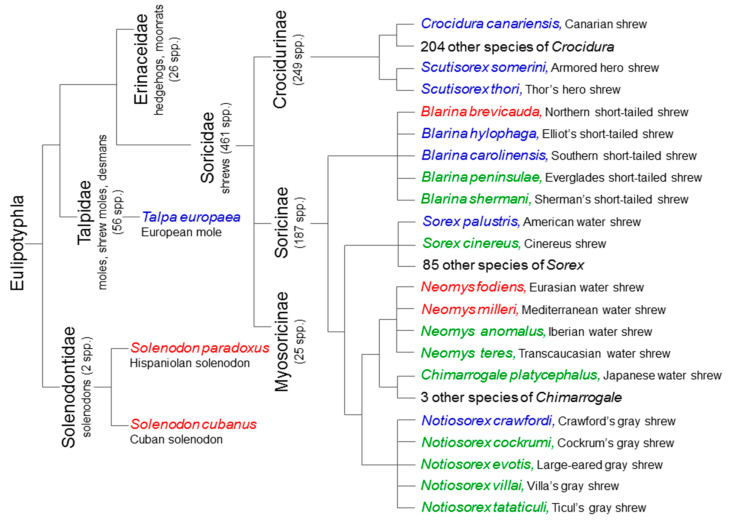
Relatedness between extant venomous eulipotyphlans. Recognized (based on toxicological and biochemical assays) venomous Eulipotyphla are shown in red. Presumably venomous eulipotyphlans (based on their feeding ecology, symptoms observed in attacked prey such as immobilization or paralysis, and presence of enlarged submandibular glands) are marked in blue. Shrews suspected of being venomous based solely on their close relationship with recognized venomous eulipotyphlans are shown in green. Phylogenetic relationships between Eulipotyphla taxa according to Dubey et al. [54]. The numbers of species are taken from the Mammal Diversity Database (https://mammaldiversity.org) visited on 14 February 2021.

**Figure 2:**
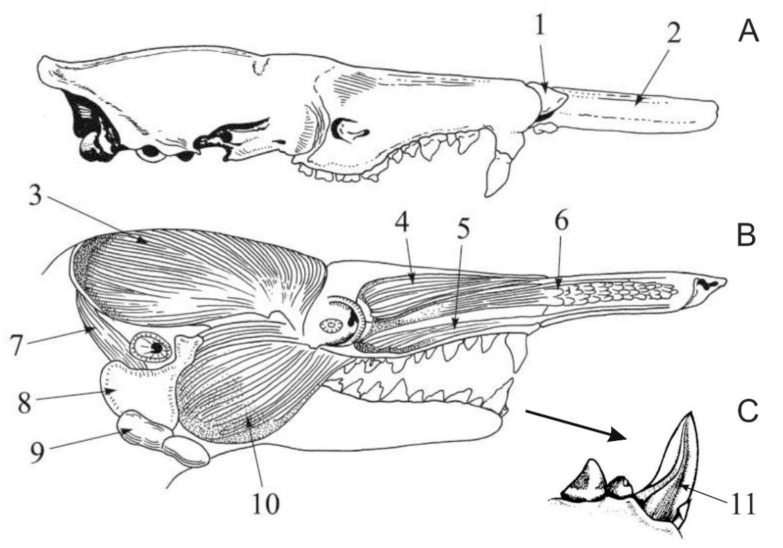
Head of the Hispaniolan solenodon (*Solenodon paradoxus*): (**A**) skull visible in profile, (**B**) head with selected muscles and salivary glands shown, and (**C**) lower incisors and canine (lingual view). 1—Os proboscidis, 2—snout cartilage; muscles: 3—temporal, 4—proper levator of the upper lip, 5—zygomatic, 6—levator of the upper lip and whiskers, 7—digastric, 10—masseter; salivary glands: 8—parotid, 9—submandibular; 11—groove in I_2_, through which toxic saliva is transported. Reproduced with permission from the Scientific Publisher PWN and Izabella Łaniecka (author of the drawing), Rząd: owadożery—Eulipotyphla *in:* Błaszak C. (ed.), Zoologia, tom 3, część 3. Ssaki; published by the Scientific Publisher PWN, Warsaw, 2020 [78], modified (part C added, redrawn from [34]).

**Figure 3:**
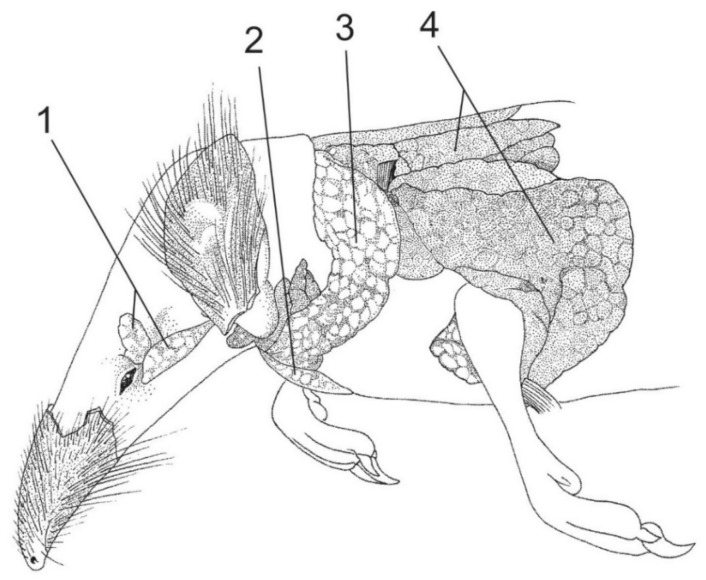
Glands of the common shrew (*Sorex araneus*), similar to those in the Eurasian water shrew (*Neomys fodiens*). 1—lacrimal gland, 2—submandibular salivary gland, 3—parotid salivary gland, and 4—brown adipose tissue. Reproduced with permission from the Scientific Publisher PWN and Izabella Łaniecka (author of the drawing), Rząd: owadożery—Eulipotyphla *in:* Błaszak C. (ed.), Zoologia, tom 3, część 3. Ssaki; published by the Scientific Publisher PWN, Warsaw, 2020 [78].

**Figure 4:**
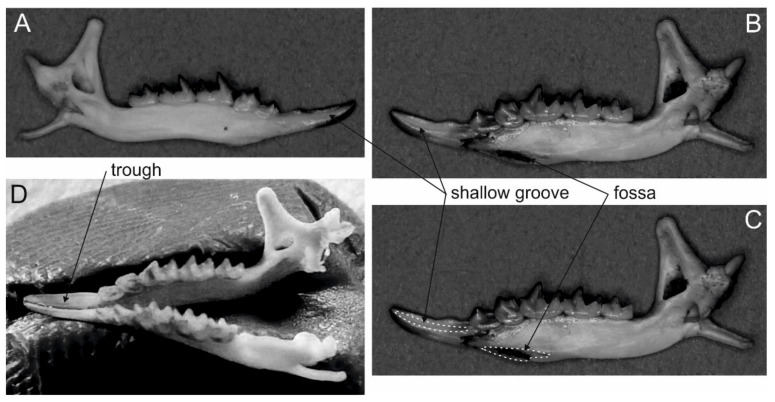
Mandibles of water shrews (*Neomys*): (**A**) left mandible of *N. milleri* and (**B**) right mandible of *N. fodiens* with shallow grooves in I_1_ and fossae in mandibles visible, (**C**) the same mandible of *N. fodiens* with the groove and the fossa demarcated with a dashed line, (**D**) two mandibles of *N. milleri* forming a trough between first incisors, which help to transport toxic saliva.

The eulipotyphlans include the majority (five confirmed and 18 suspected species) of known extant venomous mammals [34,35,36]. Nevertheless, venomousness seems to be very rare among eulipotyphlans (and mammals in general)—according to Folinsbee [58] it occurs in less than 2% of extant species. Additionally, even if future studies confirm the toxicity of saliva in the 18 suspected species, venomous species will still only make up ca. 4% of Eulipotyphla (i.e., 23 out of 545 species—see Figure 1). All representatives of this group produce venom in enlarged and granular submandibular salivary glands (Figure 2 and Figure 3) [23,59,60]. They also possess grooved teeth connected to the glands which act as a venom apparatus [23,34], enabling them to inject the toxic saliva into the target species. Solenodons (*Solenodon*) have enlarged caniniform second lower incisors I_2_ with a deep tubular channel on the anterolingual surface that widens and opens at the base of tooth ([61]; Figure 2, Table 1). In contrast, shrews (*Blarina*, *Neomys*) possess only a shallow and open groove along the lingual side of their first lower incisors I_1_. These elongated and forward-facing incisors in shrews form a concave trough (Figure 4), enabling administration of the venom into the body of prey [23,34]. It should be noted, however, that nonvenomous shrews (e.g., *Crocidura russula* and *Sorex araneus*) have a similar shallow groove in I_1_ [29]. 

Two solenodons, the Hispaniolan solenodon *S. paradoxus* and the Cuban solenodon *S. cubanus*, and three shrews—the American short-tailed shrew *B. brevicauda*, the Eurasian water shrew *N. fodiens*, and the Mediterranean water shrew *Neomys milleri*—are recognized venomous species based on toxicological and biochemical assays of their salivary gland secretions (Figure 1) [16,17,30,43,44,45,61,62,63,64,65,66]. Four other species of *Blarina* (*B. carolinensis*, *B. hylophaga*, *B. peninsulae, B. shermani*) and two species of the genus *Neomys* (*N. anomalus* (according to the latest genetic research [67,68,69], water shrews, previously classified as one species, *Neomys anomalus*, should be divided into two species: *N. anomalus* from the Iberian Peninsula and *N. milleri* from the rest of the range) and *N. teres*), each closely related to the confirmed venomous eulipotyphlans, are likely also venomous but their venoms have not yet been surveyed [35,36,59,70,71,72,73]. Preliminary studies suggest that the Canarian shrew *Crocidura canarienis* produces toxic saliva [74]; however, it would be the only venomous species of the genus Crocidura (most speciose taxa among Soricidae), and neither the composition nor toxicity of its venom have yet been examined.

Similarly, there are some observations suggesting that the masked shrew *Sorex cinereus*, the American water shrew *Sorex palustris*, two species of hero shrews (*Scutisorex somereni* and *S. thori*), the desert shrew *Notiosorex crawfordi* (and four other species of the genus *Notiosorex*: *N. cockrumi*, *N. evotis*, *N. tataticuli*, *N. villai*), the European mole *Talpa europaea* [17,35,36,75,76,77], and the Japanese water shrew *Chimarrogale platycephalus* (S. Ohdachi pers. comm.) are also presumably venomous. *Sorex palustris*, for instance, has been observed feeding on a larval Pacific giant salamander and a sculpin, and these prey were seized by the head and appeared to be immobilized [75]. Pearson [60], however, reported that saliva of this species is not toxic. Observations of foraging behavior of captive *N. crawfordi* showed that scorpions and lizards are paralyzed with the first bite, perhaps due to the toxins present in this shrew’s saliva [77]. Notably, the toxicity of saliva from this desert shrew species has also not yet been studied. Additionally, the European mole was postulated to be venomous based on the presence of large and granular submandibular glands, along with storing earthworms in a comatose state in its burrows [34]. 

However, when hunting worms, moles cut off the head segments (with nerve ganglia) of prey so that the prey remains paralyzed until full recovery [79,80]. Thus, the paralysis may result from damage to the earthworm’s nervous system, not the action of the toxic saliva. Much more research effort is therefore required to demonstrate that European moles (as well as the abovementioned shrews) produce toxic substances in their salivary glands.

## 3. Extinct Venomous Eulipotyphlans

Quite a few recently discovered extinct eulipotyphlans (both fossil and recently extinct) had grooves or channels in their teeth similar to those described above in extant venomous eulipotyphlans. Therefore, they are considered by many researchers as venomous. The fossil taxa include three species of the giant shrews (*Beremendia fissidens*, *B. minor* and *B. pohaiensis*), two species of water shrews (*Neomys newtoni* and *N. browni*), *Dolinasorex glyphodon*, *Lunanosorex lii*, and *Siamosorex debonisi*—all of which were found in Eurasia. Other recently extinct taxa inhabited the Caribbean region, including two species of solenodons (*Solenodon arredondoi* and *S. marcanoi*) and nine species (or even 12 species according to some scientists) of the genus *Nesophontes*, belonging to the family Nesophontidae, which are closely related to solenodons [25,26,27,28,58,61,81]. 

Supposed venomousness of the abovementioned species is based on (i) their relatedness to extant venomous Eulipotyphla and (ii) presence of special teeth provided with grooves enabling delivery of venom from salivary glands. In each taxon, grooves extend along the entire length of the tooth, i.e., from the base to the apex. However, there are some differences in their dental envenomation apparatus (Table 1). Most taxa (*Beremendia*, *Neomys*, and *Dolinasorex*) had one shallow, open groove along the lingual side of the first lower incisors I_1_. These elongated and forward-facing incisors formed a concave trough. The dental apparatus of *Lunanosorex lii* was almost the same but with the difference that there were two grooves on I_1_: one along the lingual side and a second along the buccal side [82]. *Siamosorex debonisi* had one deep but open groove on the mesiolingual side of the second lower incisors I_2_. These incisors were enlarged and caniniform, so they did not form any trough [26]. *Solenodon arredondoi* and *S. marcanoi,* in comparison, possessed enlarged caniniform second lower incisors I_2_ with a deep tubular channel on their anterolingual surface. It was formed by the tooth enamel and opened at the base [61,83,84]. In contrast, all species of the genus *Nesophontes* had two open grooves in the upper robust canines C^1^: one deep and wide on the anterior side, and a second deeper but narrower on the anterior-lingual side [61,83]. Therefore, dental morphology of *Nesophontes* species is unlike any extinct or living venomous taxon of Eulipotyphla (Table 1). However, it is similar to the dentition of extant helodermatid lizards from Arizona and Mexico [61] as well as an extinct eutherian *Bisonalveus browni* (order Cimolesta, related to carnivorans) from Canada, which had a wide, deep groove along the anterior side of its upper canines [24]. Nonetheless, the close phylogenetic relationship of *Nesophontes* to solenodons coupled with *Nesophontes*’ deeply grooved upper canines suggest it was venomous [58].

In addition to taxonomic relatedness with other venomous Eulipotyphla and the possession of grooved teeth, Folinsbee et al. [84] provide one more indicator of the venomousness of extinct taxa, which is the formation of a special cavity (enlarged fossa) in the mandible at the symphyseal region (Figure 4). Such a fossa occurs in most of the extant and extinct venomous eulipotyphlans (Table 1), although it is smaller and shallower in *Blarina* and *Neomys* than in, for example, the large, extinct *Beremendia fissidens* [25]. Its postulated function is to create a stronger, more immobile junction that increases bite strength [25]. This assumption is supported by the more recent study of Bennàsar et al. [85], who suggested that *Beremendia fissidens* may have had the capacity to bite prey larger than itself, such as moles (*Talpa* sp.).

These recent discoveries of extinct and presumably venomous taxa, coupled with their global distribution, suggest that in the past there were many more venomous, primarily insectivorous, mammals, which for some reason became extinct [29,34,61,81]. In addition, there are researchers who contend that some of the early Mesozoic mammals might also have been originally venomous [24,86,87]. Folinsbee [58], in contrast, claims that (i) since venomous mammals are now very rare, they were also rare in the past, and that (ii) the ability of venom production is a newly acquired feature that evolved among Eulipotyphla recently and at least three times independently [58]. The author also criticizes the inferences of widespread venom production in extinct eulipotyphlans on the basis of grooved teeth, because grooves are also present in teeth of some nonvenomous bats, primates, pigs, and carnivorans. An alternative explanation for the presence of dental grooves in these nonvenomous mammals is that they may serve to mechanically strengthen the tooth [58,88]. Thus, caution should be applied when using the presence of grooved teeth to infer the venomous nature of extinct Eulipotyphla.

## 4. Toxicity of the Eulipotyphlan Venom

The physiological effects of extracts from submandibular glands of eulipotyphlans (*Blarina*, *Neomys* and *Solenodon*) have been examined mainly on laboratory animals, i.e., mice, rabbits and cats, and rarely on wild mammals, such as voles *Microtus* sp. [34,35,36]. The symptoms were usually similar across these different mammalian taxa, with a sequence of general depression, respiratory disorder, paralysis and convulsions. The effects, however, strongly depended on the dosage and route of administration, with intracerebral and intravenous injections being far more effective than intraperitoneal and subcutaneous ones [34,62,63,64,89,90,91,92].

Pearson [89] tested the submandibular gland extracts of *B. brevicauda* on mice, rabbits and cats. Subcutaneous administration of the extract into mice caused (within one minute) immediate irritation and inflammation, followed by a general depression, locomotor impairments, eye protrusion, convulsions, respiratory failure and eventually death. After intraperitoneal injections, the symptoms were similar to those described above but without an intense local reaction. Lower doses (LD_50_ value of 150 mg/kg) were required for fatal results when compared to subcutaneous injection, and the paralysis of the mouse hindquarters appeared faster. Death of an animal occurred within 1–2 hrs. Intravenous administration also produced similar symptoms, with breathing disturbance, with protruding eyes and convulsions being the most pronounced effects. However, the effects appeared more rapidly and dramatically, with an LD_50_ of about 22 mg/kg causing death of the mouse within 20 min. For partially purified submandibular extract of *Blarina*, an LD_50_ value of 3.4 mg/kg, administered intravenously, was required to kill the mouse [90]. After intravenous injection of *Blarina* extract to a rabbit, death occurred in 5 min with an LD_50_ value of 7 mg/kg. The partially purified venom, delivered intravenously, was lethal at dose of 1.5–2.0 mg/kg [90]. Administration of 7 mg/kg of *Blarina* gland extract into the femoral vein of a cat caused respiratory disturbance but was not lethal, with recovery achieved after 15 min. A second injection of 2.2 mg/kg of extract gave similar but less pronounced effects. A third dose (7 mg/kg) revealed the same effects but the recovery was not sustained and the animal died as a result of respiratory disorder and heart failure [89]. In cats, the venom of *Blarina* caused some antiadrenalin action (which should help to overcome larger prey) as well as appeared to be a lachrymator [93].

First attempts to determine toxic effects of venom of the water shrews (*Neomys* sp.) were made by Pucek [63,64,91]. She injected intraperitoneally, intravenously and intracerebrally the saline extract of submandibular glands of *N. fodiens* into mice (*Mus musculus*), voles (*Microtus agrestis*), and rabbits (*Oryctolagus cuniculus*) [63]. Intracerebral (mice and voles) and intravenous (rabbits) injections produced the strongest and most pronounced effects. The venom of *N. fodiens* mainly affected the nervous system, as the most obvious symptoms were paralysis of the limbs and hindquarter of the body, spasms, convulsions and the loss of reactions to external stimuli. Respiratory failure and disturbances of the vascular system (a drastic drop in blood pressure) were also reported. Voles were more sensitive to *Neomys* venom than mice. The minimal dose generating a response was 0.01 mg/20 g for *M. agrestis* but 0.22 mg/20 g for *M. musculus*. The minimal LD_50_ for voles was 0.2–0.4 mg/20 g of *Neomys* gland extract, whereas for mice it was 0.5–1.0 mg/20 g [63]. To produce similar symptoms in rabbits, 20 mg/kg of *Neomys* gland extract was required, with death ensuing within 50 min. 

The saline extract of salivary glands of *N. anomalus* generated similar symptoms to those mentioned above; however, the effects were not as pronounced as in the case of extracts of *B. brevicauda* and *N. fodiens*. As reported by Pucek [64], toxic activity of gland extract of *N. anomalus* is about half as strong as that of *N. fodiens*. For instance, to kill a mouse by intracerebral administration, 1.0–2.0 mg/20 g of *N. anomalus* venom was required, whereas for *N. fodiens* it was 0.5–1.0 mg/20 g.

**Figure 5:**
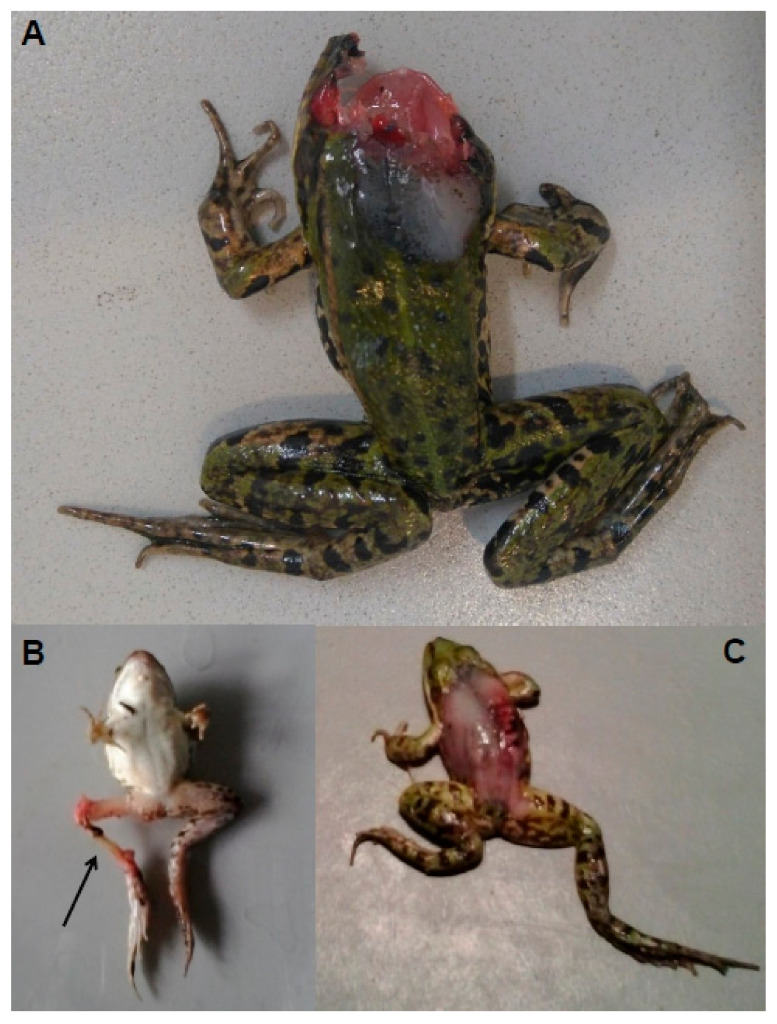
Frogs overpowered by *Neomys fodiens*: (**A**) with the head bitten, (**B**) with a leg gnawed (an arrow indicates the tibiofibula) and (**C**) with the skin removed from the frog’s back. Reproduced with permission from the Oxford University Press, Journal of Mammalogy; published by the Oxford University Press, 2018 [17].

Recently, Kowalski et al. [43] analyzed in vitro toxicity of *N. fodiens* venom on tissues from two experimental models: a beetle (*Tenebrio molitor*) and frogs (*Pelophylax* sp. and *Rana temporaria*) [94,95]. It is worth emphasizing that both beetles and frogs are natural prey of the water shrew [96,97,98,99,100,101,102]. The authors measured cardiotoxicity and paralytic activity of the whole gland extract and separated fractions from *N. fodiens* saliva on three different tissues: semi-isolated hearts of *T. molitor* and frogs; the isolated calf muscles and sciatic nerves of frogs. Venom samples with a protein concentration of 1 mg/mL were applied to all these tissue types. Their results confirmed strong paralytic (a high decrease in the conduction velocity of the frog sciatic nerve and a decrease in the force of frog calf-muscle contraction) and lower cardioinhibitory (a decrease in the frog heart contractility) properties of venom of the water shrew. Most fractions from the *Neomys* venom produced a positive chronotropic effect on the beetle heart. One fraction, however, caused a strong decrease in the contractility of *T. molitor* heart coupled with reversible cardiac arrest. Thus, these results demonstrated toxic activity of *N. fodiens* venom that may disrupt normal processes of potential prey. Behavioral tests, however, did not confirm the paralytic activity of *N. fodiens* venom on frogs, as the frogs showed no symptoms of paralysis or immobilization when being predated by water shrews [17]. Nevertheless, *N. fodiens* was able to overpower and kill frogs in laboratory tests (Figure 5) as well as in nature [97,98]. Earthworms, on the other hand, seemed to be paralyzed, thus it is likely that the venom enables the water shrew to effectively prey upon invertebrates and store them for later consumption as previously reported for *B. brevicauda* [66]. 

Gland saline extracts of *Solenodon* caused similar effects to those of shrew venom when administered to mice [62]. However, the *Solenodon* venom is 1/20 as toxic as venom of *B. brevicauda* [34]. Intraperitoneal injection of about 1 g/kg of saline extract was fatal to a mouse within 13 min, but half of the dose was not lethal within 12 hrs. Intravenous administration caused a stronger and faster response. The mouse died within 2–6 min when the gland material was injected at a level of 450 mg/kg [62]. Elevated body temperature, inflammation, thorax and head pain, and hypalbuminuria have been reported by researchers (even after prompt medical treatment) who received bites from *S. cubanus* [103]. Casewell et al. [44] found that solenodon venom displays serine protease activity and potently activates plasminogen. Administration of a sublethal dose (25 mg/kg) of venom to mice did not produce any changes in the pulse rate, respiration rate, or percentage oxygen content of the envenomed mice when compared to control animals. However, the blood flow was reduced in the envenomed animals, results confirming that solenodon venom causes hypotensive effects in vivo. This hypothesis was also supported by in vivo cardiovascular assays, in which solenodon venom (1 mg/kg) potently lowered the mean arterial pressure of anesthetized rats [44]. Casewell et al. [44] also analyzed the neurotoxicity of solenodon venom on nicotinic acetylcholine receptors (nAChRs) of human and locust (*Schistocerca gregaria*), and human voltage-gated sodium channels (Na_v_), which are commonly targeted by toxins to cause paralysis [21,33]. Solenodon venom displayed no activity on either human or locust nAChRs, but showed low but significant inhibitory activity on mammalian voltage-gated sodium channels. However, when in vivo neurotoxicity assays on locusts and centipedes (*Ethmostigmus rubripes*) (potential invertebrate prey of solenodons) were performed, no apparent symptoms of paralysis, such as immobilization or incapacitation, were observed. Therefore, the authors concluded that the recorded activity of *S. paradoxus* venom on Na_v_ channels may be specific to vertebrates [44].

Summarizing our knowledge about the toxicity of eulipotyphlan venoms studied so far, we can state that the venom of *B. brevicauda* is 3 times stronger than the venom of *N. fodiens*, 6 times stronger than the venom of *N. anomalus* and 20 times more potent than the venom of *S. paradoxus*, as well as that voles are more sensitive to the eulipotyphlan venom than mice [34,63,64].

## 5. Biochemistry of the Eulipotyphlan Venom 

Animal venoms and poisons usually consist of a complex mixture of toxins such as proteins and peptides, enzymes, amino acids, neurotransmitters, nonprotein compounds, and salts [21,33], with protein-like components usually being the most abundant. For the reasons previously outlined in Section 1.4, obtaining sufficient quantities of mammalian venom for biochemical analyses has been challenging [16,35,104,105,106]. Thus, pharmacological assays of mammalian (particularly eulipotyphlan) venoms have typically been performed using homogenized material from the salivary glands [62,63,64,89] in which toxins are produced [23,59,60]. Ellis and Krayer [90] partially purified extract from the salivary glands of *B. brevicauda*, but toxic components were not identified. In his review, Dufton [34] listed peptides and proteins of parotid and submandibular gland secretions of mammals, including monoamine oxidase. With the advent of modern venomics, this partial catalog of venom components in mammals has expanded. Table 2 summarizes what is currently known about the primary components and toxic effects of Eulipotyphla venoms. More detailed accounts of some of these findings are provided below.

In 2004, blarina toxin (BLTX), a toxic compound of the saliva of *B. brevicauda*, was purified and characterized by Kita et al. [30]. BLTX is a glycosylated protein composed of 253 amino acids with a tissue kallikrein-like protease activity. This toxin cleaves kininogens to kinins, such as bradykinin, a common mediator of inflammation, which increases vascular permeability and lowers blood pressure. BLTX administered to mice caused irregular respiration, paralysis and convulsions, and finally death. Therefore, these kinins are thought to be the main toxic agents of the eulipotyphlan venom responsible for symptoms such as dyspnea, hypotension and hypokinesia, recorded previously in pharmacological studies [30]. Mode of action of BLTX, however, still remains unknown. In 2005, Kita et al. [107] identified blarinasin, a second component of the saliva of *B. brevicauda*, which is composed of 252 amino acids and also displays a tissue kallikrein-like protease activity. However, despite a high sequence similarity to BLTX, blarinasin is not toxic to mice, suggesting that minor differences may be responsible for the toxicity of BLTX [107]. 

Another toxic component identified in the venom of the short-tailed shrew is soricidin, a 54-amino acid peptide [108], which inhibits the transient receptor potential of vallinoid type 6 (TRPV6) calcium channels [109]. Two shorter peptides, SOR-C13 and SOR-C27, derived from the C-terminus of soricidin, showed high antagonistic affinity to human TRPV6 channels that are upregulated in a number of cancers, such as ovarian and prostate tumors. Thus, these peptides provide a great opportunity to develop diagnostic and therapeutic agents helpful in cancer treatment [109]. 

Recently, Hanf and Chavez [45] also confirmed the presence of BLTX and soricidin in the venom of *B. brevicauda*. Moreover, they identified five new candidate toxins, i.e., a novel KLK1 serine protease paralog (KLK1-BL2), phospholipase A2 (PLA2), antileukoproteinase (SLPI), hyaluronidase PH-20 protein (HYALP), and a tissue factor pathway inhibitor 2 protein (TFPI2). They also identified nontoxic blarinasin and two additional KLK1 paralogs (KLK1-BL1 and KLK1-BL3) that are also not likely to be toxins as revealed by their 3D-protein structural model. SLPI was the most abundant toxin in *Blarina* saliva, followed by BLTX, the newly identified toxins KLK1-Bl2 and PLA2, and proenkephalin, which contains the known toxin peptide soricidin. Abundance of nontoxic blarinasin was also high. HYALP and TFPI2 were not expressed at high levels in the transcriptome but were relatively abundant in the saliva of the shrew. Hyaluronidase is a nontoxic protein but is a hemorrhagic spreading factor for toxins in venomous lizards [110]. TFPI2 is an important inhibitor of blood coagulation [111]. Thus, HYALP and TFPI2 appear to be important components of *Blarina* venom that may effectively contribute to its toxicity. Intriguingly, because the venom of *B. brevicauda* consists only of seven toxins, a rather simple composition given the broad diet of the short-tailed shrew, the authors speculate that the use of venom by eulipotyphlans may be of recent evolutionary origin [45]. Additionally, it is possible that other unidentified toxic compounds may be present and contribute to the toxicity of the venom of *B. brevicauda*, and some of them are likely to act synergistically with BLTX [45].

Recently, Kowalski et al. [43] identified protein-like components from the venom of *N. fodiens*. Among recognized peptides, lysozyme C, phospholipase A2 (PLA2), coagulation factor VIII, lactyloglutathione lyase and hyaluronidase may be particularly important in the toxicity of *N. fodiens* venom. Lysozyme C, which is involved in antimicrobial defense [48,112], has been previously reported in saliva of the water shrew [34]. PLA2 is widely distributed among elapid and viperid snake venoms [113,114]. This toxin displays various toxic effects, such as cardio- and neurotoxicity, and pro- and anticoagulant activity [113,114]. Thus, it is possible that PLA2 is responsible for the cardiotoxic and paralytic properties of the *N. fodiens* venom. Additionally, coagulation factor VIII may act as an antihemophilic factor [115], whereas lactyloglutathione lyase is involved in inflammation [116]. Finally, hyaluronidase, which is a common component of animal venoms [117,118], may promote the spreading of other toxins present in the *N. fodiens* venom [118,119]. Kowalski et al. [43] also found kallikrein 1-related peptidase in the saliva of the nonvenomous common shrew *Sorex araneus*. As kallikrein-like proteins seem to be widespread in the eulipotyphlan venoms, it is likely that KLK-1 paralogs (similar to BLTX) will be found in the venoms of other shrew species, including *N. fodiens*. 

Proteomic analysis of venom of the endangered Hispaniolan solenodon revealed that its venom consists of multiple paralogous kallikrein 1 (KLK1) serine proteases, with hypotensive activity. Various other protein types were also identified [44]. Comparative analyses of venoms of shrews and solenodons provide convincing evidence that eulipotyphlan venom systems have evolved convergently [44] (Table 2).

## 6. Ecological Functions of the Eulipotyphlan Venom

**Figure 6:**
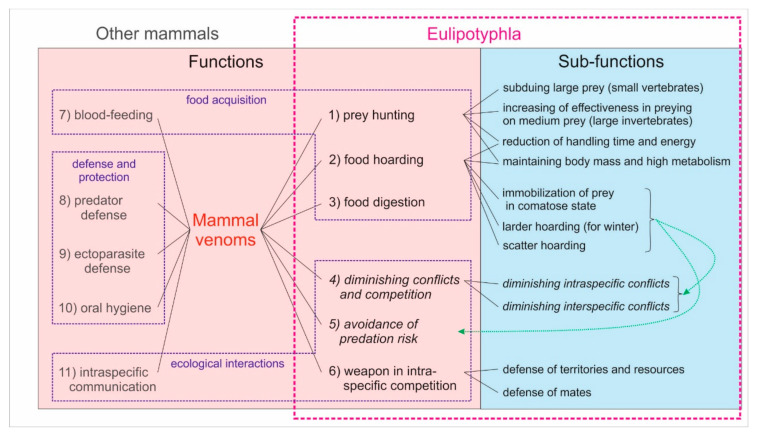
The ecological functions of venom in eulipotyphlans and other mammals, along with a number of possible subfunctions for eulipotyphlans. The functions are grouped according to three ecological pressures (food acquisition, ecological interactions, and defense and protection) that can be considered the main drivers of the evolution of venomousness in mammals. The functions given in italics (4 and 5) indirectly result from venomousness (i.e., they are derived from food hoarding and staying in shelters, as indicated by the green dotted arrows), whereas the other functions directly result from the use or action of the venom.

Interest in the evolution and ecological functions of venomousness in animals has increased markedly during the last decade [16,53,58,61,120,121,122,123]. Recently, Schendel et al. [123] distinguished 14 different functions of animal venoms, although only six were associated with mammals. However, based on the papers by other authors [16,36,53,58,61,120,121,122,124,125], and including the functions proposed below by us, as many as 11 functions of mammal venoms can be considered (Figure 6). Of these, prey hunting, predator defense, and intraspecific competition are the most frequently mentioned functions by different authors, followed by prey immobilization, ectoparasite defense and intraspecific communication [15,16,36,58,123,124,125]. The evolution of these functions and their differentiation resulted from different needs and were affected by various ecological pressures. We can distinguish at least three such ecological pressures (food acquisition, biotic interactions, and defense and protection) for venomous mammals and assign them different functions (Figure 6). These pressures can be considered the main drivers of the evolution of venom functions, but also of the differentiation of venom composition and properties. For example, defensive venoms usually have simple compositions and produce intense, localized pain, whereas venoms related to food acquisition (e.g., hunting) evolved to have mainly paralyzing activity and to become more complex, so they show a broad range of toxicity (although not always very potent) to diverse target species (prey) [123]. Moreover, this complexity increases with the breadth of the trophic niche of a venomous predator [126,127,128,129]. In turn, the venoms associated with ecological interactions (e.g., intraspecific communication or competition) should be species-specific, or even sex- or age-specific, in composition, so that they carry specific information (similarly to pheromones) and cause appropriate changes in behavior (e.g., stimulate a mating partner to copulate) [123]. The functions related to food acquisition, however, seem to be the most common and important in the majority of mammals and other animals [16,33,58,123]. 

The function of predator defense is unlikely in eulipotyphlans. They are preyed upon by large fish, frogs, reptiles, and especially birds of prey and owls. They are also killed by carnivorous mammals (such as weasels, foxes or cats), although rarely eaten because of the repelling smell of secretions from their scent glands, and not because of their venom [130,131,132,133,134,135]. However, there are some carnivores (including raccoon dogs, badgers, minks) that frequently eat shrews despite their smell [136,137,138,139]. On the other hand, eulipotyphlan venoms are weak [17,43,63,64,89,90,91] and they do not cause immediate, intense pain in humans (L. Rychlik, pers. obs. on *Neomys* species), so they are unlikely to be able to effectively incapacitate or deter predators such as owls, foxes or cats. As far as we know, there are no observations or even suggestions that venom protects eulipotyphlans against ectoparasites or helps them maintain oral hygiene (e.g., by reducing the number of bacteria), although some venom components from *Blarina* and *Neomys* saliva exhibit antimicrobial activity (Table 2). None of the known extant Eulipotyphla feeds on blood in the way that vampire bats do, so the function of blood-feeding (*sensu* Schendel et al. [123]) may also be excluded in this order.

Nevertheless, we suggest there are at least six functions of eulipotyphlan venom, including a number of different subfunctions (Figure 6). Some of these functions are currently hypothetical, but others have empirical support. It should also be remarked here that these functions are not mutually exclusive, so a species may derive several different benefits from its venom. 

Here are presented the possible functions of the venom in Eeulipotyphla, starting with those related to food acquisition:

(1) **Prey hunting**—venom facilitates prey capture. This function can be divided into several subfunctions:

(a) Venom enables subduing of relatively large prey such as small-sized vertebrates, which was suggested, among others, by Dufton [34], Rychlik [140] and Rode-Margono and Nekaris [16]. This possibility is confirmed by numerous data on natural diets of venomous shrews and solenodons, which include small vertebrates and other relatively large prey, and direct observations of their attacks on such prey. Hunting and eating of large prey have been especially often observed in *Blarina* [65,132,141] and *Neomys fodiens* [97,98,142,143]. 

There is evidence that *B. brevicauda* eats vertebrates such as salamanders, snakes, small birds, small mammals, and even small hares [132,144]. This shrew hunts effectively on rodents, especially young ones [141,145,146,147]. Rood [148] reported 10 out of 12 mice killed by a short-tailed shrew in less than 20 min. Hence, some authors even suggest that shrew predation can influence population dynamics or space use by rodents [141,149,150]. Also *B. carolinensis* is able to kill and eat young rodents of three wild cricetid species [70].

Many authors [96,97,98,101,131,142,143,151,152,153] reported *N. fodiens* preying on frogs, tadpoles, newts and fish, both under laboratory conditions and in the wild. Dufton [34] cites an example of one *N. fodiens* that ate a bird (bullfinch). According to Brehm (cited in [154]), in captivity, water shrews killed fish up to 60 times heavier than itself. In our experiments, *N. fodiens* (but not the nonvenomous *S. araneus*) was able to overpower and kill frogs [17]. 

*Crocidura canarienis* is able to hunt and immobilize Atlantic lizards (*Gallotia atlantica*) [74], and *Notiosorex crawfordi* is able to paralyze and subdue some invertebrate and lizard species larger than itself [77].

For solenodons, it is known that in addition to invertebrates and plant food, they eat small terrestrial vertebrates (lizards, snakes, frogs, and birds) and their eggs. Moreover, there are big arthropods (e.g., crabs) and those equipped with chemical defenses (e.g., scorpions, centipedes, and millipedes) among the prey eaten by these species [34,35,155,156]. In captivity, solenodons actively pursued, killed, and ate mice and chickens [34,155].

In all of the abovementioned examples, subjugation of prey could be greatly facilitated by injecting venom into their body. The use of venom is also supported by observations that while attacking salamanders or frogs, *B. brevicauda* and *N. fodiens* bit the head and neck regions of their prey ([65,96,101,157] and L. Rychlik, pers. obs.), seemingly to inject the venom into or close to the brain. Similarly, *B. carolinensis* killed young mice by a bite to the base of the skull [70]. However, it was also observed that *B. brevicauda*, attacking a snake, bit every part of its body [144], whereas in our recent experiments, frogs were principally bitten by water shrews in fore- and hind-limbs and sides of the body [17].

(b) Relatively weak venom of eulipotyphlans (see above Section 4) increases effectiveness in hunting medium-sized prey (i.e., large invertebrates) rather than large-sized prey (i.e., small vertebrates). This idea was suggested by a few authors [17,58,66] and is supported by the fact that venomous eulipotyphlans eat mainly invertebrates, not vertebrates (e.g., [66,99,102,132,156]). Similarly, the extinct and presumably venomous shrews of the genus *Beremendia* probably mainly hunted beetles and snails, i.e., medium-sized invertebrates [29]. *Blarina brevicauda* uses venom as an immobilizing agent for snails [158] and insects [66,159], with the immobilizing effect of its venom being stronger on insects than on anurans [160,161]. We found that venom of *N. fodiens* had stronger cardioinhibitory effects on contractility of the insect heart than on the frog heart [43]. Consistent with this, the weak venom of this species was helpful in overpowering medium-sized prey (earthworms) but not so in overcoming large prey (frogs) [17]. Additionally, time needed from the first bite by *N. crawfordi* to the death of its prey was much longer for lizards than for invertebrate prey [77]. Lastly, nonvenomous *S. araneus* needed at least twice as much time as *N. fodiens* to kill large beetles and was not able to overpower the largest ones that were killed and eaten by *N. fodiens* [151]. *Sorex araneus* also required significantly more time than *N. fodiens* to subdue earthworms of proportionally similar sizes [17]. All of these examples suggest that eulipotyphlan venoms may have evolved, at least in part, to prey on large invertebrates.

(c) Venom shortens handling time and/or helps to save energy required to overcome prey, as suggested by Dufton [34] and Rode-Margono and Nekaris [16]. The support for this subfunction comes from our experiments: *N. fodiens* required significantly less time than *S. araneus* to subdue prey of proportionally similar sizes and this difference grew with increases in prey size [17]. Similarly, in Haberl’s [162] experiments, the handling times of mealworm larvae were shorter for venomous water shrews (*N. fodiens* and *N. anomalus*) than for nonvenomous species (*Sorex araneus*, *S. minutus*, *Crocidura suaveolens*), with the differences, depending on the interspecies comparison, ranging from 3.2 to even 33.9 s for handling a single larva. 

All of these observations, along with many examples of eulipotyphlans hunting large prey given above under the subfunction 1a), suggest that venom may enable them to reduce handling time and costs, and to gain more energy per unit of foraging time. Such efficient foraging is especially important for Soricinae shrews which, due to their extremely high metabolic rates, tight energy budget and huge food requirements [163], must forage particularly effectively, because wrong foraging decisions may lead to their death in a short time.

Another beneficial aspect of using venom while attacking large and potentially dangerous prey is to reduce the risk of retaliatory injuries that could be inflicted by such prey if they were not, more effectively, incapacitated or paralyzed by the action of the venom [34]. Such a function is proposed to be one of the drivers of venom evolution in snakes [34,164].

(d) Venom enables large shrews to maintain their body mass and high metabolism, which was suggested by Folinsbee [58]. She notes that venomous *Blarina* and *Neomys* have both large body mass and high metabolic rates. Hence, Folinsbee [58] argues that “There may be selective pressure on a trait like venom, which enables large shrews to collect more prey in order to maintain their mass and high metabolism. Smaller shrews, even with high [basal metabolic rates] BMRs, do not need to consume as many calories, and may therefore be capable of storing sufficient food without the need for venom”. Indeed, most of the venomous or suspected venomous shrews are relatively large (*Chimarrogale* and *Scutisorex* are even larger than *Blarina* and *Neomys*), but this idea has not been systematically investigated.

(2) **Food hoarding**—venom helps to make food stores. This function is manifested in several subfunctions, and generates two indirect functions, diminishing conflicts /competition and avoidance of predation risk (see below).

(a) Venom allows eulipotyphlans to cache food composed mainly of large prey, postulated, for example, by Rychlik [140] and Rychlik and Jancewicz [165]. This possibility is based on numerous experimental observations that shrews usually consumed smaller prey (e.g., fly larvae, mealworms, other small insects) immediately when captured but hoarded larger prey items (e.g., crickets, roaches, snails, fish, frogs, mice, and voles) or food portions [66,140,157,165,166,167,168]. For example, an individual of *B. brevicauda* cached as many as 56 ca. 3 cm-long frogs in a large food store [157]. Additionally, in nature, the food stores of venomous shrews usually included large prey [158,168,169]. Hoarding large prey rather than small ones may be advantageous because: (i) transport of one (or a few) large prey items to the shelter is usually less energetically costly than multiple transports of small prey; (ii) large prey items provide a food supply for a longer time and remain fresh longer than small items [65,140,165,166]. Given that venom permits eulipotyphlans to subdue large prey, being venomous has the added benefit of facilitating efficient food storage. However, this subfunction of venom is to a certain extent undermined by observations that nonvenomous shrews (e.g., *Cryptotis parva*, *Sorex minutus*, *S. araneus*) also mainly hoard large prey and eat small prey immediately upon capture ([17,165,170] and L. Rychlik and P. Kardynia, unpubl. data).

(b) Venom paralyzes or immobilizes prey that can be hoarded fresh (live, in a comatose state) for later consumption. This subfunction was suggested by Martin [66] and is accepted by other authors [16,17,34,121]. Martin [66] has shown that the short-tailed shrew uses venom to paralyze its hoarded prey (crickets and roaches) and commented that if the collected insects were dead, many of them would lose substantial nutritive value before the shrew could eat them. Hoarding of comatose prey (including snails, insects or mice) by *B. brevicauda* was also observed in other studies [65,158,159,166]. Similarly, *N. fodiens* immobilized (or, as the author writes, “semi-paralyzed”) its prey such as earthworms, large slugs and sticklebacks [96]. Cranbrook [96] also observed that if worms paralyzed by *N. fodiens* are given suitable conditions and time, they recover from a water shrew’s bite. This, according to the author, indicates the paralysis is caused by toxic effect of saliva and not by mere mechanical damage. In our experiments [17], water shrews also immobilized and hoarded earthworms. 

However, water shrews overpowered earthworms in a way that we classified as mechanical immobilization (i.e., by many and frequent bites distributed along the whole body of the prey which could lead to damage of its nervous system) rather than by venomous paralyzing (in fewer bites injecting venom directed to the head region of prey) [17]. Additionally, the role of venom in hoarding prey in a comatose state is undermined by observations of hoarding immobilized prey by nonvenomous shrews. For example, *Sorex bendirii*, *S. pacificus* and *S. araneus* immobilized earthworms and other large invertebrates with rapid series of bites along their bodies and stored them, and the prey remained alive even for over 20 h [17,171,172]. Therefore, the possible subfunction of eulipothyplan venom in providing stores of paralyzed but otherwise fresh prey would benefit from more research.

(c) Paralytic venom helps eulipotyphlans in food-storing for winter (i.e., in larder hoarding). Such a possibility was suggested by several authors [16,66,121,168,173], who state that hoarding of live but comatose prey may be especially advantageous in cold seasons when food supplies are reduced in both quantity and quality. This is supported by the finding that food hoarding by short-tailed shrews occurred primarily in autumn and winter [166]. Similarly, *N. fodiens* (but also nonvenomous *S. araneus*) hoarded significantly more food (per capita and per unit of body mass) in winter than in summer [174]. Moreover, it was observed in winter under natural conditions that *B. brevicauda* stored snails and then took care of its stores, i.e., carried them to the ground surface when it was cold, returning the snails to the burrow when the temperature rose, probably to keep them fresh longer (Shull 1907 cited by [169]). Thus, venom-facilitated food hoarding may be an important part of a wintering strategy because it enables shrews to remain in their warm nests for longer during periods of cold weather [35,168,173].

(d) Venom may also help in scatter hoarding. This subfunction is based on foraging experiments where venomous shrews hoarded prey/food in scattered hiding places (*B. brevicauda*—[157,175]; *N. anomalus*—[140,167]; *N. fodiens*—[153,176]). Possibly, thanks to the venom, shrews can quickly subdue many prey items and hide them in caches scattered across their home ranges. 

(e) Venom-facilitated food hoarding is profitable for shrews that cannot store much energy in their bodies in the form of adipose tissue. We suggested such an advantage as even large Soricinae shrews (e.g., *Blarina* and *Neomys*) have high metabolic rates, but low ingestion capacity and small energy reserves [163,177,178] and thus may be sensitive to food shortages [179]. In short, venom-mediated food hoarding may behaviorally compensate for physiological limitations on energy storage [178,180,181].

(3) **Food digestion**—venom contains enzymes that help in food digestion. Lawrence [159] and Pournelle [23] suggested the proteolytic enzymes from venom may help initiate digestion of the large volume of proteins consumed by venomous shrews. The need for consumption of high quantities of protein-rich prey, and thus for having powerful digestive enzymes in the saliva, could arise from the generally high metabolic demands of Eulipotyphla [16,34,163]. However, so far there are no data supporting this function.

(4) **Diminishing conflicts and competition** with both con- and heterospecifics—this is an indirect function resulting from venom-facilitated hunting and prey hoarding rather than the use of venom in direct interactions between animals. It also has a few subfunctions: 

(a) The venom-facilitated hoarding of prey (especially large items) enables food consumption in shelters. This, in turn, allows eulipotyphlans to stay longer or leave the shelter less often to acquire food. In consequence, shrews may be exposed to fewer contacts with competitors, which should diminish competition [65,140,165,182,183].

(b) By hunting large prey (such as vertebrates), venomous shrews acquire more nutritious food portions than nonvenomous species, but also reduce the competition with them for smaller (invertebrate) prey [34]. In particular, venom may enable semiaquatic shrews to hunt larger aquatic prey, and thus to avoid or diminish competition with shrews preying on smaller terrestrial prey. This idea was suggested by Churchfield [184], but her findings on natural diets of semiaquatic and terrestrial shrews did not support this [184]. However, later, Churchfield and Rychlik [102] found that terrestrial and nonvenomous *Sorex* shrew species ate more small prey (≤5 mm) than did semiaquatic and venomous *Neomys* species. Similarly, Rychlik and Jancewicz [165] found in their experiments that (i) *N. fodiens* hoarded 3–5 times heavier prey than nonvenomous *Sorex araneus* and *S. minutus*, and (ii) both venomous water shrew species (*N. fodiens* and *N. anomalus*) hoarded small fish in a high proportion (in contrast to the two *Sorex* species). On the other hand, nonvenomous pigmy shrews (*S. minutus*) hoarded and ate significantly smaller prey (mainly larvae of terrestrial flies and mealworms) than the three larger species [165]. In line with this, large shrews display some specialization and preference to hunt large prey, and small shrews show specialization and preference for small prey items in the wild [182,185,186].

(c) Venom-facilitated scattered food caching may be profitable for shrews that cannot defend resources against larger competitors. In comparison to larger predators, even large shrews are so small that they are not able to defend their food resources against most competitors [178,179,180]. Thus, scatter hoarding may be advantageous because it “minimizes the loss of food to con- and heterospecifics by increasing the dispersion of the resource, making it less efficient for a potential competitor to steal from a hoard than to forage” [170]. Therefore, the scattered food caching is expected among shrews [165,179,187] and venomousness can greatly increase the effectiveness of this behavior, which in turn should contribute to the reduction in inter- and intraspecific competition.

(5) **Avoidance of predation risk**—this is also an indirect function resulting from feeding on cached prey (subdued with venom) in shelters (i.e., venom is not used directly to deter or defend against predators). Hoarding of prey allows shrews to leave their shelters less often to acquire food and thus to reduce their predation risk (which is important for small mammals such as shrews, which themselves can be prey to larger predators) [65,140,165]. In particular, hoarding of immobilized prey enables larger shrews to take long diurnal breaks in activity. Such a function has been suggested by Maser and Hooven [172] for nonvenomous *Sorex pacificus* as an adaptation to its mainly nocturnal activity. Thus, since *Neomys* shrews also display the unimodal nocturnal activity pattern with low activity during the day [188] and are small enough to be prey of many predators, their hoarding of immobilized prey might be explained as for *S. pacificus* [17]. This function can be also supported by the fact that smaller and nonvenomous shrews (such as *Sorex vagrans*, *S. araneus*, *S. minutus*) are active throughout the 24 h cycle [172,188] probably because they hoard less durable food stores and/or consume them faster.

(6) Venom as **weapon in intraspecific competition**—this function was suggested by Rode-Margono and Nekaris [16] and Ligabue-Braun [121] for solenodons. It is based on Rabb’s [62] observation of high mortality among Hispaniolan solenodons kept together in enclosures, whose only visible wounds were the bite marks by conspecifics on their feet. However, there is also ample evidence of interspecific competition [102,184,188,189,190,191,192,193,194] and high interspecific aggressiveness among shrews, with venomous species usually dominating these interactions [195,196,197,198,199,200]. Larger venomous shrews were even observed to kill smaller shrews ([141,201,202] and L. Rychlik pers. obser.). High interspecific aggressiveness is typically motivated by a need to defend mates or/and territories with food (including food stores) or other resources [16,53,183,199], and meeting all of these needs may be more effective by using venom in intraspecific conflicts (Figure 6).

However, Casewell et al. [44] state that “solenodons are relatively social animals; both species live in family groups comprising adults, subadults, and young, with multiple family groups of Cuban solenodons sharing the same den”. They also argue that “although a lack of natural history reports documenting the behavior of these poorly known mammals limits our interpretation, we find no convincing evidence supporting the hypothesis for venom having evolved for an intraspecific purpose”. Moreover, there are suggestions that solenodons (as well as *Blarina*) are immune to their own toxins [23,34,62]. 

The importance of venom during intraspecific fights among eulipotyphlan competitors also seems unlikely due to their high metabolic rates. Each direct interaction of this kind, with the use of venom and with wounds requiring healing, would constitute an unnecessary waste of energy. Therefore, the well-developed system of vocal and olfactory communication of these mammals, especially shrews, seems to be a more advantageous and sufficient solution, allowing for the exchange of information between individuals and avoiding combat [36,203].

One last possibility should be mentioned, namely that the ability to produce venom may be an ancestral legacy, which may currently have no function or give no benefit in extant eulipotyphlans. This option was considered by Dufton [34] and Folinsbee [58]. However, in our opinion, this seems unlikely due to the possibility that venom production by eulipotyphlans is probably metabolically costly, as it is in snakes [204,205]. Given such costs and in the absence of any current utility, selection should have favored individuals with mutations leading to a reduction or an arrestment in venom production. Such adaptive venom loss has been already reported for some fish-egg eating snakes [206].

## 7. Why Are So Few Eulipotyphlans Venomous?

Venom production is very rare among extant eulipotyphlans: it occurs in only ca. 1% of species currently recognized as being venomous, increasing to 4% if species suspected of being venomous are included (compare Section 2 and Figure 1). According to some authors [24,34,81,86,87], venomousness may be an ancestral trait, which was more common among early than among modern mammals, and has been preserved in only few extant eulipotyphlans. In contrast, Folinsbee [58] and Arbuckle [122] claim that venomousness evolved more recently and several times independently but only in a few eulipotyphlans (and mammals in general) and, thus, is characteristic of only a few extant species. Regardless of which of these evolutionary paths is true, a question arises as to why venomousness is so rare, since it can provide many adaptive functions.

Several hypotheses have been proposed [58,121] to explain this rarity among extant and extinct Eulipotyphla: (1) As previously mentioned, the production and application of venom may not be an adaptive trait, i.e., it does not provide benefits either in defense or in food acquisition. (2) There are certain biological (e.g., morphological or physiological) limitations to the production of venom. (3) Venom production is so expensive that it is more profitable to invest energy in less costly mechanisms of defense or hunting. (4) The production and use of venom is profitable only for a few species with a specific biology or ecology. (5) It can be also explained by “the concept of past over-predation” which was proposed for *Solenodon* species by Dufton [34], who writes that due to their venomousness, solenodons could become “victims of their own success”. This means that early *Solenodon* species (and other eulipotyphlans) lost the capacity to produce venom because they were too successful as predators and drove their vertebrate prey extinct. This resulted in a shift to an insectivorous diet in early eulipotyphlans, with a subsequent venom loss in almost all species [34]. (6) Mammals (including eulipotyphlans) have evolved faster methods to subdue prey, using “tooth and claw” rather than relying on the slower method based on venom injection [34,125]. This may have been selected for because of the faster pace of life and much higher metabolism of mammals compared to reptiles [163,207]. Snakes (e.g., rattlesnakes, vipers) are able to survive at least several months without food [208,209] so, after biting prey, they can easily wait additional 5–15 min before eating it. In contrast, predatory mammals with a comparable or smaller body mass (e.g., weasels or insectivores) can survive without food for only several hours or a day, so postponing the consumption of prey even a few minutes may be too risky for them. This is especially true of the Soricinae shrews, which feed every 1-2 h and die after 3–4 h without food ([163] and L. Rychlik, pers. obs.). In addition, snakes do not have limbs and claws that could help them incapacitate and handle prey. Therefore, the ability to paralyze or kill prey with a potent venom before swallowing has been favored more strongly in snakes than in mammals [34].

As an alternative to these six hypotheses mentioned above, it is possible that the number of extant venomous eulipotyphalns is, in fact, much higher, especially among shrews (cf. Figure 1 and Section 2), but so far the vast majority of species have not been thoroughly investigated for venom. We take sides of this alternative.

## 8. Conclusions

Venom has evolved multiple times throughout the animal kingdom, but is rare amongst mammals [16,58,122,123]. Most venomous mammal species belong to the order Eulipotyphla (see Section 2) [36,121]. The need for venom production likely results from the high metabolic demands of eulipotyphlans, requiring a high rate of prey acquisition with minimal energy expenditure, and who risk retaliatory damage while attacking (particularly with larger and more difficult to subdue prey) [34]. As in other venomous predators, venom may help eulipotyphlans acquire larger energy portions (in the form of medium and large prey items) and reduce handling time or costs (through quicker overpowering prey), functions confirmed by both toxicological and behavioral studies [17,43,63]. Moreover, venom may also help eulipotyphlans in food hoarding, and especially in making long-term food stores, because it enables hoarding prey in a comatose state. If so, then food hoarding can additionally save energy and time spent on prey searching and catching, as well as minimize the risk of predation and conflicts with competitors (by utilization of food stores in shelters) [140,182,183,210,211,212]. Nevertheless, these mutually nonexclusive functions of venom, as well as the biochemistry, genetic basis of venom production and possible occurrence of venomousness in other eulipotyphlan species, are still poorly investigated and deserve more attention. Knowledge about factors shaping the ecological functions of venom will enhance our understanding of the ecology and evolution of venomous eulipotyphlans which, in turn, may help in their conservation.

## Figures and Tables

**Table 1:** Dental morphology related to envenomation apparatus and location of venom-secreting salivary glands in extant and extinct eulipotyphlans. Genera of extant taxa marked with “?” include species only suspected of being venomous. All extinct taxa are supposed (but not proved) of being venomous. References are given in the text.

Taxon	Grooved Teeth	Number, Form and Position of Grooves	Lower Inci-sors Forming a Trough	Enlarged Fossa in Mandibles	Venom-Secreting Salivary Gland
**Extant eulipotyphlans**					
Shrews (*Blarina*, *Neomys*, *Chimarrogale*?, *Crocidura*?, *Notiosorex*?, *Scutisorex*?, *Sorex*?)	I_1_	one shallow groove, open, lingual side	yes	yes	submandibular
Solenodons (*Solenodon*)	I_2_	one deep groove forming a channel, anterolingual side	no	yes	submandibular
Moles (*Talpa*?)	no	none	no	?	submandibular?
**Extinct eulipotyphlans**					
Shrews *Beremendia fissidens, B. minor* and *B. pohaiensis*	I_1_	one shallow groove, open, lingual side	yes	yes	submandibular
*Neomys newtoni* and *N. browni*	I_1_	one shallow groove, open, lingual side	yes	yes	submandibular
*Dolinasorex glyphodon*	I_1_	one narrow but conspicuous groove, open, lingual side	yes	yes?	submandibular
*Lunanosorex lii*	I_1_	two grooves, open, lingual and buccal sides	yes	yes	submandibular
*Siamosorex debonisi*	I_2_	one deep but open, mesiolingual side	no	no	submandibular
Solenodons *Solenodon arredondoi* and *S. marcanoi*	I_2_	one deep groove forming a channel, anterolingual side	no	yes	submandibular
Nesophontids *Nesophontes* (~9 species)	C^1^	two open grooves: deep and wide on anterior side, deeper and narrow on anterolingual side	no	no	parotid?

**Table 2:** Primary components and toxicity of eulipotyphlan venoms.

Species	Venom Components	Venom Activity	References
American short-tailed shrew *Blarina brevicauda*	blarina toxin (BLTX)	proteolytic and hypotensive activity	[30]
	blarinasin	nontoxic	[107]
	soricidin	inhibition of the movement of Ca across the cellular membrane	[108,109]
	kallikrein 1 (KLK1-BL2) serine protease	hypotensive effects in vivo	[45]
	phospholipase A2 (PLA2)	cardio-, myo- and neurotoxicity, pro- and anticoagulant effects	[45]
	antileukoproteinase (SLPI)	inhibition of serine-proteases, antimicrobial activity	[45]
	hyaluronidase PH-20	facilitation of toxin spreading	[45]
	tissue factor pathway inhibitor 2 protein	inhibition of blood coagulation	[45]
Eurasian water shrew *Neomys fodiens*	phospholipase A2 (PLA2)	paralytic effects cardiotoxic activity in vitro	[17,43]
	hyaluronidase	facilitation of toxin spreading	[17,43]
	lysozyme C	antimicrobial defense	[17,43]
Hispaniolan solenodon *Solenodon paradoxus*	kallikrein 1 (KLK1) serine protease	hypotensive effects in vivo	[44]

## Data Availability

Not applicable.
